# Rapid seroconversion and persistent functional IgG antibodies in severe COVID-19 patients correlates with an IL-12p70 and IL-33 signature

**DOI:** 10.1038/s41598-021-83019-0

**Published:** 2021-02-10

**Authors:** Ariel Munitz, L. Edry-Botzer, M. Itan, R. Tur-Kaspa, D. Dicker, D. Marcoviciu, M. G. Goren, M. Mor, S. Lev, T. Gottesman, K. Muhsen, D. Cohen, M. Stein, U. Qimron, N. T. Freund, Y. Wine, Motti Gerlic

**Affiliations:** 1 Department of Clinical Microbiology and Immunology, Sackler School of Medicine, Tel Aviv University, 6997801 Ramat Aviv, Israel; 2 Department of Medicine D and the Liver Institute, Rabin Medical Center, Beilinson Hospital, Molecular Hepatology Research Laboratory, Felsenstein Medical Research Center Sackler School of Medicine Tel-Aviv University, Faculty of Medicine Bar-Ilan University, Liver Institute Rabin Medical Center Beilinson Hospital, 39100 Petah Tikva, Israel; 3 Internal Medicine D, Hasharon Hospital-Rabin Medical Center, Petach Tikva, Israel; 4 Sackler School of Medicine, Tel Aviv University, 6997801 Tel Aviv, Israel; 5 Intensive Care Unit, Hasharon Hospital-Rabin Medical Center, Petach Tikva, Israel; 6 Infectious Diseases and Infection Control, Hasharon Hospital-Rabin Medical Center, Petach Tikva, Israel; 7 Department of Epidemiology and Preventive Medicine, School of Public Health, Tel Aviv University, 6997801 Tel Aviv, Israel; 8 Allergy and Clinical Immunology Unit, Wolfson Medical Center, 6997801 Holon, Israel; 9 The Shmunis School of Biomedicine and Cancer Research, The George S. Wise Faculty of Life Sciences, Tel Aviv University, 6997801 Tel Aviv, Israel

**Keywords:** Infectious diseases, Viral infection

## Abstract

Despite ongoing efforts to characterize the host response toward SARS-CoV-2, a major gap in our knowledge still exists regarding the magnitude and duration of the humoral response. Analysis of the antibody response in mild versus moderate/severe patients, using our new developed quantitative electrochemiluminescent assay for detecting IgM/IgA/IgG antibodies toward SARS-CoV-2 antigens, revealed a rapid onset of IgG/IgA antibodies, specifically in moderate/severe patients. IgM antibodies against the viral receptor binding domain, but not against nucleocapsid protein, were detected at early stages of the disease. Furthermore, we observed a marked reduction in IgM/IgA antibodies over-time. Adapting our assay for ACE2 binding-competition, demonstrated that the presence of potentially neutralizing antibodies is corelated with IgG/IgA. Finally, analysis of the cytokine profile in COVID-19 patients revealed unique correlation of an IL-12p70/IL33 and IgG seroconversion, which correlated with disease severity. In summary, our comprehensive analysis has major implications on the understanding and monitoring of SARS-CoV-2 infections.

## Introduction

The eruption of the COVID-19 pandemic, caused by the newly discovered SARS-CoV-2 virus, has had a profound impact on human life on a global scale^1,2^. COVID-19 has affected and still affects millions of people worldwide, resulting in high mortality and morbidity rates as well as high health care costs and difficulties in treatment^3^. Furthermore, unprecedented government interventions indirectly caused significant morbidity and mortality^4^. This is exemplified by the intense engagement of most health facilities with COVID-19; consequently, making them unavailable to patients suffering from other diseases and conditions^5^. In addition, the overwhelming economic burden that COVID-19 imposes on most countries is expected to result in the loss of numerous additional lives, including health care workers, along with extensive long-term damage^6^.

Detection of infected individuals is typically carried out by using RT-PCR analysis, which amplifies viral genes. Although this method is an excellent tool for surveillance of viral spread, it has major drawbacks, including decreased accuracy when swabs are taken 5 days after symptoms onset (~ 70%)^7^. Furthermore, it is expensive and does not provide substantial data on the immunity of a given individual in the population. Thus, although excellent tools exist for the diagnosis of viral load and the diagnosis of infected individuals, a major gap still exists in understanding and effectively responding to the host. Recent studies assessed the kinetics of the humoral immune response following SARS-CoV-2 infection and associated between the emergence of different antibody subsets their titers and disease severity. Nonetheless, data from additional patient cohorts is still missing^8–11^.

Since the main hurdle in generating such knowledge is the development of reliable diagnostic tools, multiple approaches aimed to generate accurate serological testing. However, the presence of multiple asymptomatic individuals and the fact that it remains unclear whether antibodies are generated with the onset of symptoms strengthen the need for kinetic analysis of the host response using rapid and accurate serological assays. Reliable serological tests can provide critical clinical information regarding the course of the disease and the host response^12^. Finally, since mucosal tissues, such as the respiratory tract, are affected in COVID-19. Thus, it is extremely important to monitor the differential expression of Immunoglobulin (Ig) IgA titers compared to IgM and IgG antibodies and to correlate seroconversion of these Ig’s with clinical outcomes^13^.

The invaluable insights that can be achieved by serological tests prompted us to develop an accurate and sensitive assay that detects the expression and potential neutralizing activity of the three main antibody classes, IgM, IgG, and IgA, using electrochemiluminescence. We validated the assay using 104 samples from hospitalized and recovered COVID-19 patients as well as and 195 serum samples that were obtained before November 2019. Using our new developed quantitative assay, we determined seropositivity of IgM, IgG, and IgA antibodies toward the receptor binding domain (RBD) of the spike protein as well as the nucleocapsid protein (NP) of SARS-CoV-2. Furthermore, we analyzed the cytokine response and subsequently compared the cytokine and antibody response in mild versus moderate/severe patients. Finally, we modified our test to enable the evaluation of antibody neutralization potential by comparing their ability to block the binding of angiotensin-converting enzyme 2 (ACE2) to RBD. Thus, our platform can be used for two highly necessary tasks: (1) rapid assessment of total antibody titers; and (2) potential neutralization capabilities of antibodies against SARS-CoV-2. Collectively, this study provides a major technical advance and comprehensive analysis of the human antibody and cytokine response, which has major implications on our understanding and monitoring of SARS-CoV-2.

## Results

### Development of SARS-CoV-2 electrochemiluminescence-based ELISA

For developing a serological assay for SARS-CoV-2, we sought to calibrate an electroluminescence-based ELISA test according to the following criteria: First, the assay should be accurate and should display > 95% sensitivity and > 97% specificity. Second, the assay should be potentially enable multiplexing of several antibody classes toward different SARS-CoV-2 antigens. Finally, the assay should be robust so that it can be upscaled in regular hospitals and/or community health laboratories to enable the testing of multiple individuals simultaneously.

**Figure 1:**
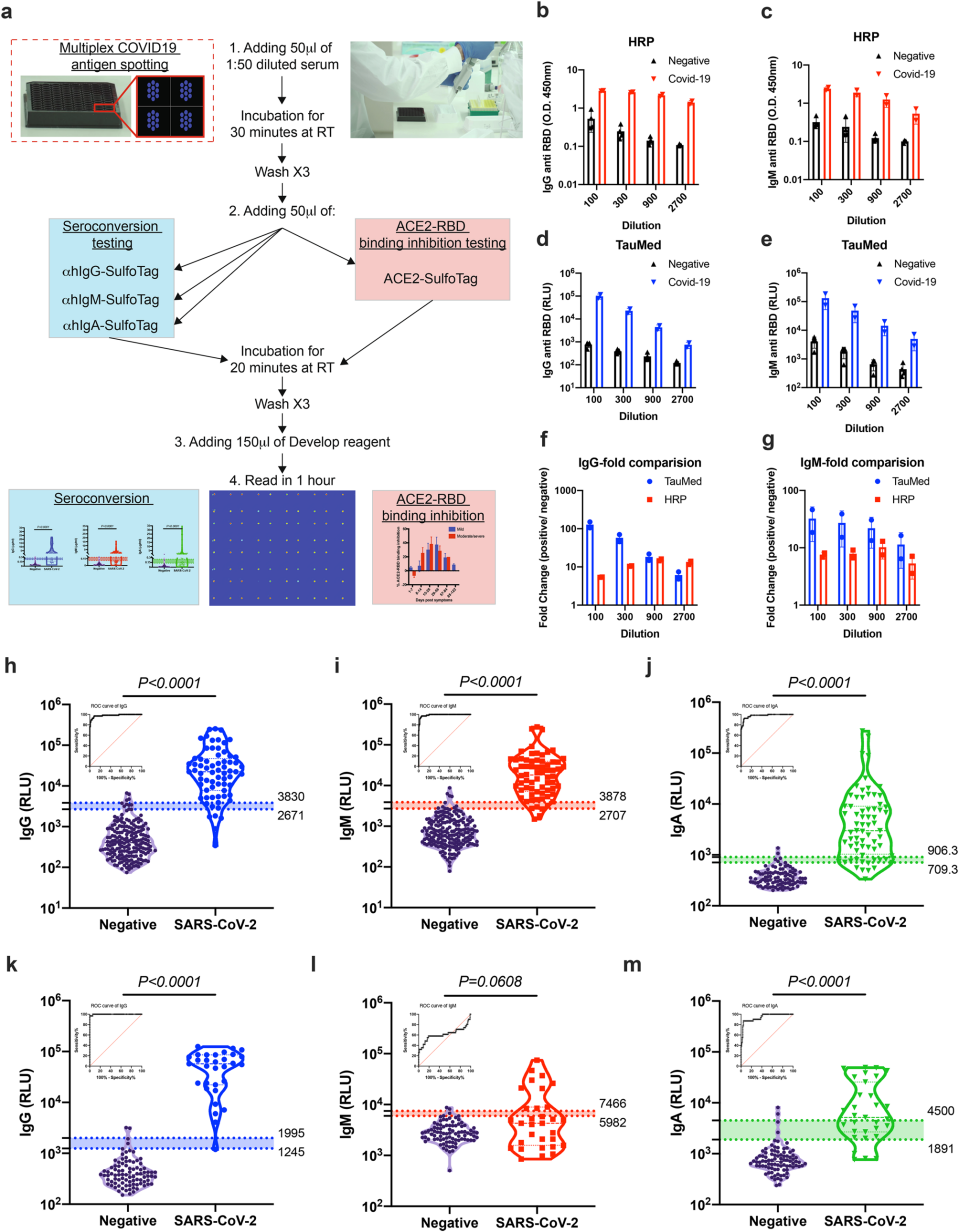
Validation of anti-SARS-CoV-2 antibodies using electrochemiluminescence ELISA. (**a**) Schematic illustration of our new developed serological test. (**b**–**g**) Comparison of SARS-CoV-2 HRP-based ELISA to electrochemiluminescence-based ELISA. The presence of anti-RBD IgG and IgM antibodies was determined in a side-by-side comparison using HRP-based ELISA (HRP) (**b**,**c**) and electrochemiluminescence-based ELISA (TauMed) (**d**,**e**). The fold changes in the values obtained from individual positive samples (n = 4) over the average negative samples (n = 2) were calculated (**f**,**g**). (**h**–**m**) Validation of anti-SARS-CoV-2 antibodies using electrochemiluminescence ELISA. Peripheral blood was collected from the peripheral blood of > 14 DPS of hospitalized COVID-19 patients and anonymous recovered patients (n = 68 and n = 31 for RBD and NP respectively). Negative samples were obtained from true SARS-CoV-2 negative patients (i.e., prior to the SARS-CoV-2 pandemic) (n = 197 and n = 90 for RBD and NP respectively). Plasma was obtained, diluted 1:50, and added to a 96-well plate precoated with SARS-CoV-2 RBD (**h**–**j**) or NP (**k**–**m**) antigens. IgG (**h**,**k**), IgM (**i**,**l**), and IgA (**j**,**m**) levels as well as ROC analysis are shown. Data were calculated using GraphPad Prism 8; the dotted line represents the calculated cutoff value discriminating between positive and negative samples. A nonparametric Mann–Whitney t-test was performed. P values are shown.

To this end, we hypothesized that an electrochemiluminescent-based assay would be an ideal platform (Fig. 1a)^14,15^. Electrochemiluminescence is a type of luminescence that is produced during electrochemical reactions in solution. Such assays provide a high dynamic range and are fully quantitative. Furthermore, by placing high binding carbon electrodes at the bottom of multi-spot microplates, such assays allow easy attachment of multiple biological reagents such as SARS-CoV-2 antigens and potentially enable high-throughput multiplexing.

First, we performed a side-by-side comparison between a standard enzymatic (i.e., horseradish peroxidase-based) ELISA test (termed HRP) and an electrochemiluminescence-based ELISA test (termed TauMed). The HRP test could detect IgG and IgM antibodies against the SARS-CoV-2 RBD (Fig. 1b,c, respectively). However, it was non-linear in the lower serum dilutions, both in IgM and IgG (Fig. 1b,c). In contrast, the TauMed test displayed a linear titration, which was observed in the diluted COVID-19-positive serum. (Fig. 1d,e). Furthermore, a dose-dependent signal-to-noise ratio was observed in the TauMed electrochemiluminescence test but not the standard HRP test (Fig. 1f,g). Finally, the TauMed electrochemiluminescence test showed a higher sensitivity (~ 100-fold for IgG and ~ 30-fold for IgM), in comparison with HRP (~ tenfold for both IgG and IgM). These results demonstrate the superiority of the TauMed assay over the HRP assay in all tested parameters.

### Validation of SARS-CoV-2 electrochemiluminescence-based ELISA

To validate our assay and determine the cutoff range for assay specificity and sensitivity, we obtained sera from hospitalized and recovered COVID-19 patients (the patient characteristics are summarized in Supplementary Table 1) and recovered COVID-19 patients, as well as sera from patients that were not exposed to SARS-CoV-2 (e.g., serum that was obtained before November 2019). A significant increase in electrochemiluminescence was observed in our patient cohort, demonstrating the presence of anti-RBD IgG, IgM, and IgA antibodies (Fig. 1h–j). Using ROC analysis of sera from > 14 days post the onset of symptoms (now termed: DPS) from COVID-19 patients and recovered individuals (n = 68) and all of the negative samples (n = 195 for IgG and IgM and n = 97 for IgA), we determined a cutoff value of ~ 95% and ~ 98% specificity (Wilson/Brown 95% CI) and the equivalent sensitivity for these cutoffs for all three antibodies (Fig. 1h–j and Supplementary Table 2). Since individual patients may test positive to one (13 out of 96) or two (25 out of 96) out of the three antibody classes (see Supplementary Table 3), we further analyzed our data using a combined IgG, IgM, and IgA strategy. In this analysis, positivity toward COVID-19 seroconversion was determined by testing positive (using the ~ 98% specificity) for only one out of three specific RBD antibody classes. This new combined analysis resulted in 94.9% specificity and increased the sensitivity from ~ 78–91% to 100% for the COVID-19 patients and recovered individuals, which were more than 14 days post symptoms. Using individual and combined approaches, we analyzed all patient samples that were also obtained at earlier time points and that had post symptoms (i.e., < 14 DPS). This approach could detect COVID-19 seropositive patients even in the early stages of the disease (Supplementary Fig. 1a,b and Supplementary Table 3). In fact, the sensitivity increased from ~ 46 to 61% for a specific antibody class to 84.6% using the combined strategy at ≤ 7 DPS and from ~ 40–73.3% to 80% in the second week of post symptoms. In total, regardless of DPS, the sensitivity increased from ~ 75–79% to 94.8%.

The specific immune response toward distinct viral antigens may result in different kinetics of the humoral antibody response^16^. Thus, we aimed to determine the presence of antibodies toward an intra-viral protein/antigen, which may be presented to the immune system at later times post-infection. This is in addition to antibodies directed against the RBD domain, which is within the SARS-CoV-2 spike protein and thus is more likely to be presented to the immune system at earlier times post-infection as suggested by higher sensitivity of IgM antibody production towards SARS-CoV-1^17^. To this end, we used the nucleocapsid protein (NP) as an additional antigen. A significant increase in anti-NP IgG and IgA antibodies (Fig. 1k,m) but not IgM antibodies (Fig. 1l) was observed in our patient cohort. Using ROC analysis of the > 14 DPS COVID-19 patients (n = 31) and negative samples (n = 90), we determined a cutoff value for achieving ~ 95% and ~ 98% specificity (Wilson/Brown 95% CI) and the equivalent sensitivity for all three antibodies (Fig. 1k–m and Supplementary Table 4). Furthermore, we employed our combined IgG, IgM, and IgA analysis strategy (Supplementary Fig. 1c,d and Supplementary Table 5). Since the NP antigen appears to elicit an antibody response that is primarily IgG, this combined analysis strategy was less efficient than what we obtained when analyzing anti-RBD antibody responses.

### SARS-CoV-2 RBD antigen as a serological marker shows superior results to NP antigen

**Figure 2:**
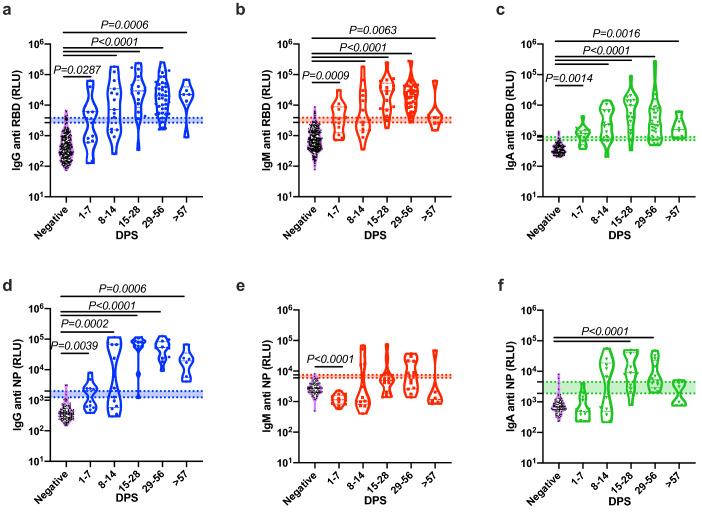
Comparison of the development anti-SARS-CoV-2 RBD and NP antibodies. Peripheral blood was collected from hospitalized COVID-19 and recovered patients. Negative samples were obtained from true SARS-CoV-2 negative patients (i.e., prior to the SARS-CoV-2 pandemic). Plasma was obtained, diluted 1:50, and added to a 96-well plate precoated with SARS-CoV-2 RBD (**a**–**c**) or NP (**d**–**f**) antigens. IgG (**a**,**d**), IgM (**b**,**e**), and IgA (**c**,**f**) levels are shown. (**a**–**f**) Kinetics of all samples. Data were calculated using GraphPad Prism 8; the dotted line represents the calculated cutoff values (95% and 98% sensitivity) discriminating between positive and negative samples. Statistical analysis was performed using a Nonparametric Kruskal–Wells test for multiple comparisons.

Next, we compared the kinetics of the host antibody response in our patient population. To this end, we divided the patients into four groups: 1–7 DPS, 8–14 DPS, 15–28 DPS, and > 29 DPS. Notably, the latter group consisted of patients with active disease and recovered individuals. As shown in Fig. 2a–c and Supplementary Fig. 2a,c,e,g all antibody classes against SARS-CoV-2 RBD antigen developed rapidly, and were readily detected even in the patient groups that were in their first days post symptoms. In contrast, IgG and IgA antibodies against SARS-CoV-2 NP antigens develop much slower (Fig. 2d–f and Supplementary Fig. 2b,d,f,h). In agreement with our hypothesis regarding the time of antigen exposure, IgM anti-NP antibodies did not develop within the first fourteen days post symptoms. On the other hand, IgG antibodies against NP reached a peak similar to that of anti-RBD, showing high specificity after two weeks.

### Combined analysis using the presence of anti-RBD and anti-NP IgG antibodies demonstrates highly specific diagnostic potential

Given that our assay offers the potential to multiplex several antigens at the same time, we decided to examine whether we could enhance our diagnostic potential by analyzing the presence of the two IgG molecules targeting RBD and NP together. In this analysis the “criteria” for positivity toward COVID-19 seroconversion was determined by testing positive for either anti-RBD or anti-NP IgG antibodies using a cutoff of 100% specificity to each individual antibody. Although increased assay specificity usually results in decreased sensitivity, this combined analysis enabled us to maintain the high sensitivity of the assay. In fact, with a specificity of 100%, we were still able to achieve 96.8% sensitivity for the > 14 DPS COVID-19 patients and recovered individuals (Supplementary Table 6).

### Rapid onset of antibodies in moderate/severe patients in comparison to mild ones

**Figure 3:**
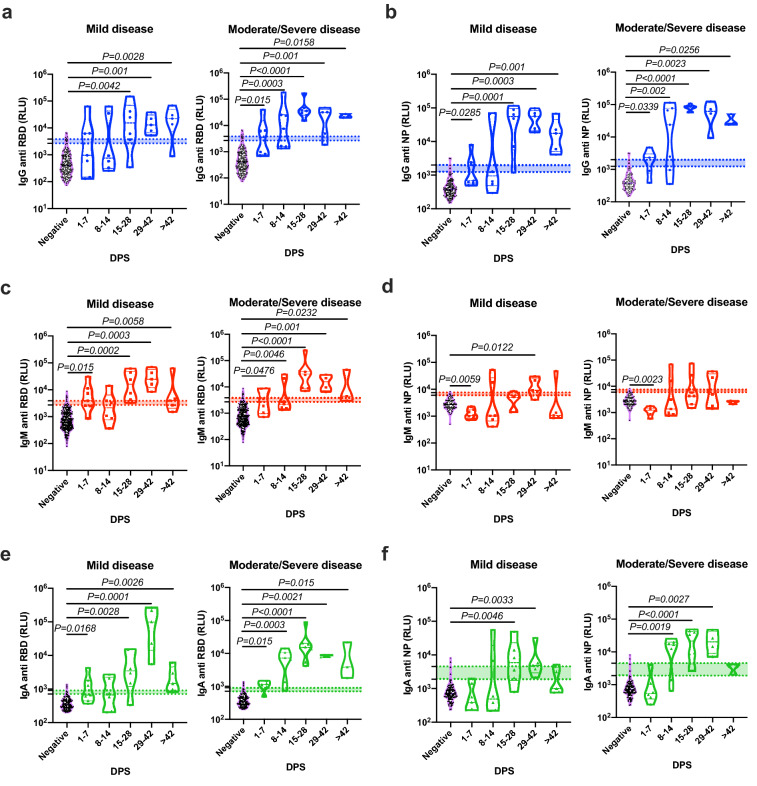
Correlation of the antibodies’ response to the disease severity. Peripheral blood was collected from hospitalized COVID-19 patients. Negative samples were obtained from true SARS-CoV-2 negative patients (i.e., prior to the SARS-CoV-2 pandemic). Plasma was obtained, diluted 1:50, and added to a 96-well plate precoated with SARS-CoV-2 RBD (**a**,**c**,**e**) or NP (**b**,**d**,**f**) antigens. Patients’ antibody results were grouped according to their disease severity and graphed against DPS. Data were calculated using GraphPad Prism 8; the dotted line represents the calculated cutoff values (95% and 98% sensitivity) discriminating between positive and negative samples. Statistical analysis was performed using a Nonparametric Kruskal–Wells test for multiple comparisons against negative samples. Significant P values are shown.

To assess whether the antibody response during SARS-CoV-2 infection correlated with clinical parameters, we divided our patient cohort into two groups consisting of mild and moderate/severe patients (see Supplementary Table 1). A pooled analysis of all the sera antibody titers, independent of disease severity, revealed that antibodies (i.e., IgG, IgM, and IgA) against RBD and NP peaked between 15 and 28 DPS. IgM against NP appeared to peak slightly later and maximal antibody titers were observed at 29–56 DPS (Fig. 2e). Notably, although still detectable, IgM and IgA antibody classes started to decrease at > 57 DPS, whereas IgG levels remained relatively stable (Fig. 2a,d). Next, the readout for each antibody class was assessed in each patient group (see Supplementary Table 1) separately (Fig. 3). This analysis revealed that the onset of the antibody response was rapid in moderate/severe patients in comparison with mild patients for IgG and IgA antibodies against both RBD and NP (Fig. 3). Despite the slower kinetic pattern, all patients, regardless of their disease severity, eventually develop similar levels of antibodies. Although not statistically significant, a trend toward reduction in IgM and IgA was observed in the late stages of the disease (i.e., > 42 DPS) in both populations of patients, whereas only a slight reduction was observed in IgG antibodies.

### Antibody kinetics and its association with gender and clinical parameters

Previous data suggested that males are more susceptible to develop severe COVID-19 disease^18^. In support of these data, the male-to-female ratio in the mild COVID-19 patient cohort was 0.81, whereas in the moderate/severe patient population it was 0.22, demonstrating the predominance of males over females in moderate/severe patients (Supplementary Table 1). Given this gender difference in our patient cohorts, we aimed to determine whether this may bias our data toward the rapid onset of antibodies in moderate/severe patients in comparison to mild ones (Fig. 3). Thus, we assessed the onset of antibodies toward RBD and NP in males vs. females in each patient cohort. No significant differences were observed between the different genders (Supplementary Fig. 3). In addition, no significant correlations were observed between any antibody response and the levels of CRP and/or lymphocyte cell counts (Supplementary Fig. 4). Furthermore, no correlation was observed between lymphocyte counts and disease severity.

### The presence of SARS-CoV-2 neutralizing antibodies correlates with anti-RBD IgG and IgA levels

**Figure 4:**
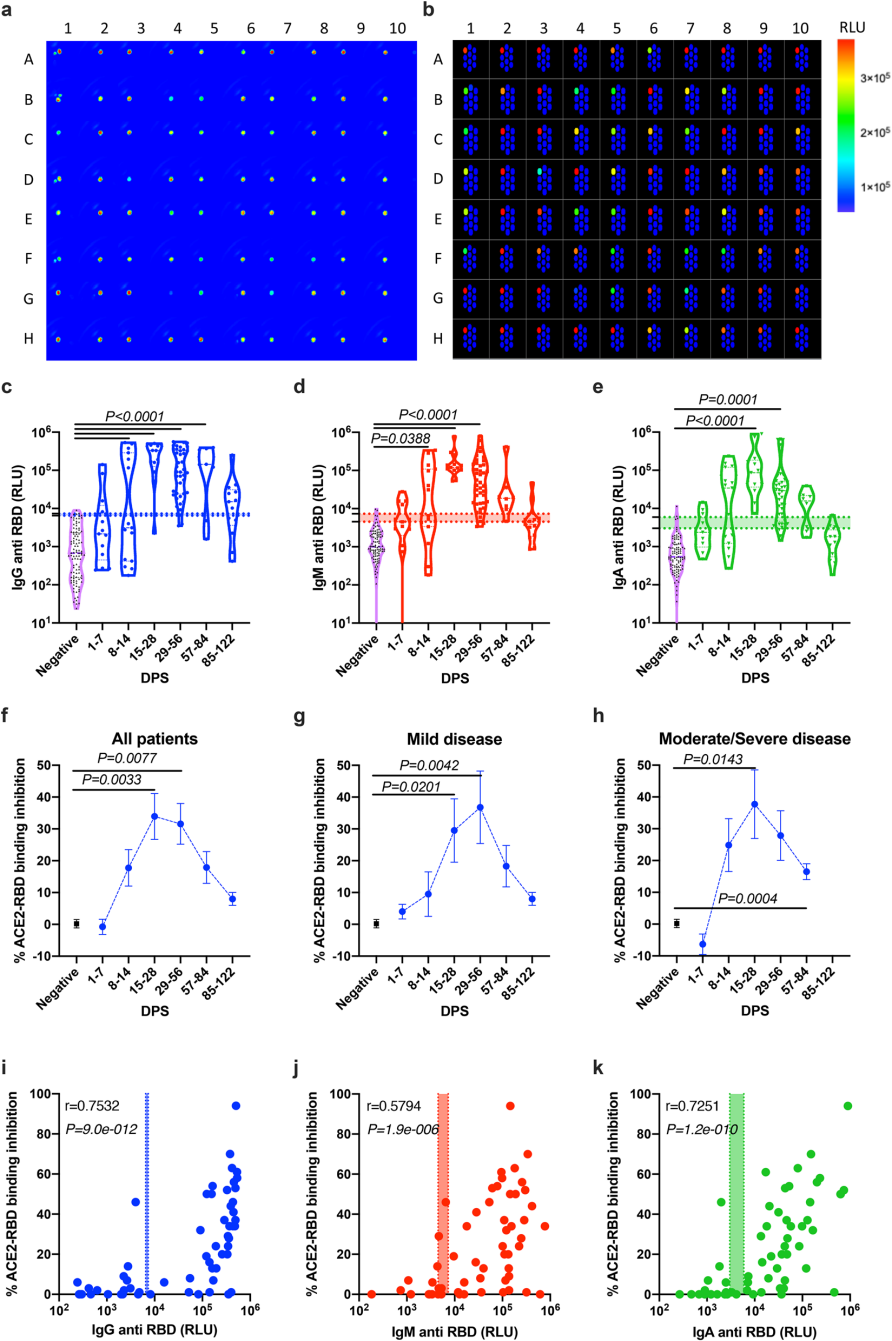
Anti-SARS-CoV-2-RBD neutralization antibodies using spotted electrochemiluminescence ELISA. Peripheral blood was collected from hospitalized COVID-19 and anonymous recovered patients (n = 75). Negative samples were obtained from true SARS-CoV-2 negative patients (i.e., prior to the SARS-CoV-2 pandemic) (n = 4). Plasma was obtained, diluted 1:50, and added to a 10-spot 96-well plate spotted with SARS-CoV-2 RBD antigen on spot number 1, and BSA on spots number 2–10. ACE2-sulfotag was used instead of a secondary/detection antibody. Representative photomicrographs of real time (**a**) and schematic (**b**) images of the spotted 96 well plate are presented. Kinetics of all patients (**c**), mild (**d**) and moderate/severe (**e**) patients is shown; average ± SEM. Inhibition of ACE2-RBD binding was calculated from the average RLU of the 4 negative plasma donors. (**f**–**h**) Patients’ antibody results were graphed against the neutralization antibody response. (**i**–**k**) Correlation analysis of antibody vs. ACE-RBD binding. Data were calculated using GraphPad Prism 8; the dotted X-line represents the calculated cutoff values (95% and 98% sensitivity) discriminating between positive and negative samples. (**c**–**e**) A Nonparametric Kruskal–Wells test for multiple comparisons. (**i**–**k**) Correlation analysis was performed using a nonparametric Spearman’s correlation test (two-tailed, 95% confidence). P values are shown.

One of the important questions regarding the antibodies, which were generated in response to SARS-CoV-2 is whether they have functional neutralizing activities. Thus, we aimed to determine whether the antibody response during SARS-CoV-2 infection correlates with the production of potential neutralizing antibodies. To this end, we first enhanced the detection capabilities of our serological assay by spotting the RBD antigen on one spot out of a 10-spot 96-Well plate (Fig. 4a,b); BSA was spotted on the remaining nine spots and was used as an internal blank control (in the future each of the BSA spotted spot can be substituted by other SARS-CoV-2 antigen). Using this new improved protocol, we were able to achieve a higher dynamic range in comparison with our original protocol where the RBD antigen was coated on the entire well (Fig. 4c–e versus Fig. 2a–c). This was observed by significantly higher signal to noise ratio for all of the three tested antibodies, namely, IgG, IgM and IgA (Supplementary Fig. 5a–f versus Fig. 1h–j). These improved dynamic range and signal to noise ratio are probably due to a stronger and better yield of RBD coating. Furthermore, using an anti SARS-CoV-1 RBD IgG1 antibody, which was shown to bind SARS-CoV-2, we were able to quantify the concentration of antibodies directed to RBD (Supplementary Fig. 5g,j–l).

SARS-CoV-2 binds to human cells via the direct interaction of S1 RBD with the human protein ACE2. Thus, inhibition of RBD-ACE2 interactions can be used to determine the neutralizing potential of a patient’s antibodies in the serum. In the next set of experiments, we assessed the ability of serum from patients to compete with the binding of ACE2 to the coated RBD using recombinant ACE2 conjugated to biotin together with Sulfo-Tag-streptavidin. The percent of inhibition observed is proportional to the neutralization potential of the COVID-19 patient’s antibodies (Supplementary Fig. 5h). Assessment of the levels of neutralizing antibodies in our entire patient cohort showed that the development of neutralizing antibodies was evident starting at 7 days post symptoms onset (Fig. 4f). Comparison of neutralizing antibody levels between mild and moderate/severe patients revealed that in moderate/severe patients, the presence of neutralizing antibodies was observed sooner (between day 8–14) than in mild patients (between days 15–28) (Fig. 4g–h). In agreement with antibody affinity maturation of 4–5 days, a delay in the development of neutralizing antibodies was observed in the first week post symptoms (Fig. 4f–h) in comparison to the total antibody response (Fig. 4c–e). Finally, the development of the neutralization antibodies was highly correlated with the presence of total IgG and IgA antibodies and to lesser extent to IgM (Fig. 4i–k).

### Cytokine profiling of COVID-19 patients reveals a unique cytokine signature that is associated with disease severity and seroconversion

**Figure 5:**
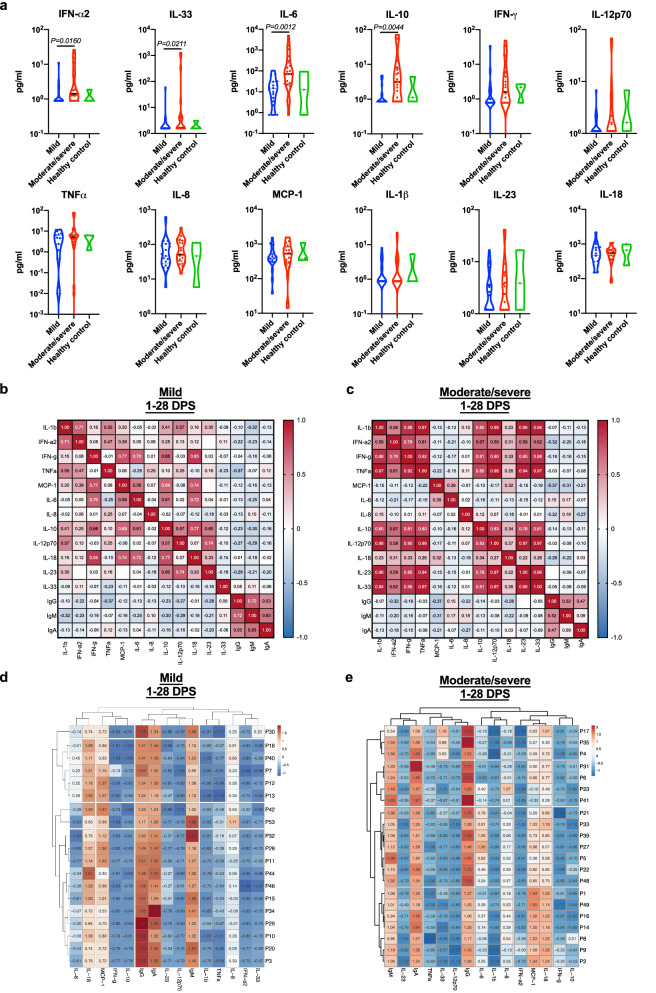
Correlation of the cytokine response to the disease severity and to the antibodies’ response. Peripheral blood was collected from hospitalized COVID-19 patients in their 30 days post symptoms (n = 40). Negative samples were obtained from true SARS-CoV-2 negative patients (i.e., prior to the SARS-CoV-2 pandemic) (n = 3). (**a**) Plasma was obtained, diluted 1:2, and cytokines analysis was performed using 13 multiplex kit, Biolegend. At least 250 beads were acquired per cytokine, using AttuneNxT. Cytokine calculation was performed using LEGENDplex v.8.0 software. A Nonparametric Mann–Whitney test was performed between mild and severe disease patients; P values are shown. (**b**,**c**) Two tailed (95% confidence) Pearson r correlation between patients’ cytokine and seroconversion toward SARS-CoV2 RBD IgG/M/A antibodies was preformed using GraphPad Prism 8. (**d**,**e**) Hierarchical clustering of patients’ cytokine and seroconversion toward SARS-CoV2 RBD IgG/M/A antibodies was preformed using ClustVis; Original values are ln(x + 1)-transformed. Rows are centered; unit variance scaling is applied to rows. Both rows and columns are clustered using correlation distance and complete linkage.

Disease severity in COVID-19 patients was suggested to be related to uncontrolled inflammation^19,20^. Furthermore, the cytokine profile following the immune response towards SARS-CoV-2 may point out specific immunological pathways (i.e., T cell-mediated immune responses), which may govern the host response^21^. Therefore, we aimed to monitor serum cytokine expression patterns in our patient cohort. We were specifically interested to determine the cytokine profile during the acute phase of disease (i.e., 1–28 DPS) where we identified rapid seropositivity and neutralization potential in moderate/severe patient in comparison to mild patients (Fig. 4c–h). Using a multiplex panel of 13 different cytokines, we identified marked elevation in the levels of IFN-α2, IL-33, IL-6, and IL-10 in moderate/severe patients in comparison with mild patients (Fig. 5a); Although a trend toward increased expression of IFN-γ and IL-12p70 was observed in moderate/severe patients, increased expression did not reach statistical significance. Next, we assessed the general correlation of cytokine and antibody response in each patient cohort (mild vs. moderate/severe disease). In agreement with significant higher cytokine expression in moderate/severe patients (Fig. 5a), a strong correlation between additional cytokines was observed in moderate/severe patients in comparison to mild ones (Fig. 5b,c). In fact, a high correlation was observed between cytokines that mediate T cell-mediated immune responses including IFN-γ, IL-12p70, TNFα (representing Th1 immunity); IL1β, IL-6 and IL-23 (representing Th17 immunity), and IFN-α and IL-18 (representing γδ-T cell immunity). A minor correlation was observed in both patient cohorts between IgG/IgM production and IL-6 expression, albeit this correlation was slightly higher in the moderate/severe patient population. Finally, we analyzed our two cohorts using a hierarchical clustering correlation (Fig. 5d,e). This analysis resulted in distinct clustering between mild and moderate/severe cohorts. While all antibody classes cluster together in the mild cohort, a separation between IgM/A to IgG was observed in the moderate/severe patients. Interestingly anti-RBD IgG in the moderate/severe cohort clustered together with IL-33.

## Discussion

Herein we describe a rapid, quantitative, accurate, and robust serological method to detect seroconversion upon SARS-CoV-2 infection and the neutralization potential of the detected antibodies. Our method is based on the reactivity of the major classes of antibodies, namely, IgG, IgM, and IgA toward the immunogenic RBD and NP proteins of the virus, and their ability to specifically inhibit the interactions of RBD with ACE2 and thus potentially neutralize viral infection (Fig. 1a). This method has several advantages over the standard ELISA procedures. For example, many standard ELISA tests, which are used for diagnostic serological testing, rely on enzymatic activity for the end point detection of the antibodies. This enzymatic activity introduces several limitations. Since the enzymatic reaction is time dependent, many ELISA tests require the use of a standard curve in order to detect the sample in the linear range of the assay. In electrochemiluminescence-based assays, such as the one we have developed, each individual sample in the plate is electronically excited and emits light, which is recorded immediately. This enables the assay to have a wide dynamic range that exceeds that of the standard ELISA tests. An additional advantage of the test we developed is the high positive-to-negative ratio. Using a standard ELISA test, we could achieve a ~ tenfold induction, whereas using our assay we reached ~ 100-fold in the homemade coated plates and more than ~ 500-fold in the spotted plates. An additional advantage of this platform is the ability to assess all three major antibody classes using multiplexing, which is an advantage even in respect to recently published serological tests^8–11^. This is especially important in diagnostics since it allows to increase the sensitivity of the assay by cross-analyzing the formation of different antibodies in each individual. Indeed, although 81.25% of patients developed at least two out of all three antibodies toward the viral RBD, 13.5% of them could generate only a single antibody class (e.g., they were positive for IgM but not for IgA or IgG). Such patients would be perceived as patients that did not develop antibodies if they were assessed by assays that enable the detection of 1 or even 2 antibodies. Strengthening this notion, our combined analysis strategy increased the assay’s sensitivity to nearly 100%. This is noteworthy, since all of our analyses were conducted using a threshold that corresponds to ~ 98% specificity to each individual antibody. For comparison, the three leading serological test, Roche Elecsys Anti-SARS-CoV-2, Abbott SARS-CoV-2 IgG and DiaSorin LIAISON SARS-CoV-2 S1/S2 IgG, which all achieved > 99% specificity, results in sensitivity of 89.2%, 84.6% and 83.1% respectively^22^. In addition, since IgG antibodies last for longer time periods post infections, a preferred option for population surveys may be to combine two or even more antigens to increase sensitivity. Indeed, our combined analysis for IgG against either RBD or NP SARS-CoV-2 antigens, result in 96.8% sensitivity for > 14 DPS without compromising the specificity of the assay, which was set on 100%. Finally, as every individual has a different background, using our quantitative and highly sensitive method can be used to monitor antibody response in individual patients regardless of the cutoff, that is used for the entire population (see an example in Supplementary Fig. 5i).

To better define the host antibody response toward different viral antigens, we compared the onset of antibody generation toward the SARS-CoV-2 antigens, RBD and NP. Our data indicate marked differences in the generation of an IgM response between these two distinct antigens. Whereas IgM antibodies toward RBD were readily detected even in early days post-symptoms (e.g. first week), IgM antibodies against NP did not develop. This is most likely explained by the kinetics of viral entry and replication in mucosal epithelial cells^13^. Initial exposure of the immune system is probably initiated by external antigens (even in a low viral load), whereas only later on, when the viral load increases and perhaps immune-mediated epithelial cell and/or viral death occurs, internal antigens such as NP are exposed. The finding that different viral antigens elicit differential kinetics in terms of the antibody’s response is important, since this may be an underlying difference between different serological testing methods, where, for example, an individual generated antibodies toward RBD but not NP^9,11,23^. This point will need further studies as other publication were able to better detect NP antigens^10,24^. Directly related, the technological basis of our assay allows each well to be coated within a given 96-well plate with several antigens (up to 10 different antigens per well). This will enable rapid multiplexing of differential antibody responses against several viral antigens within a given sample and will enable one to obtain a comprehensive understanding of the host response toward SARS-CoV-2.

In agreement with a previous publication^25^, our analysis of the antibody response in mild vs. moderate/severe patients revealed that moderate/severe patients generated a relatively rapid antibody response, especially toward RBD. Although our study bears the limitation that we do not know the time of infection and that the reference point for our analyses is the time post symptoms, these data clearly indicate that the disease severity is not due to a lack of antibody response in moderate/severe patients. In fact, our cytokine data corroborate previous publications describing increased immune cell activation and subsequently an increased pro-inflammatory response of the host in severe patients^26^. Thus, we believe that the rapid production of antibodies in moderate/severe individuals reflects this phenomenon. Further studies are required to correlate between the level of antibody response and the viral load of a given individual by obtaining the RT-PCR Ct values of each patient.

Several studies raised the hypothesis that moderate/severe COVID-19 patients may elicit a elicit antibody dependent enhancement of the immune response^27^. In this scenario, part of the antibody repertoire generated by the host activates antibodies, which can induce immune cell activation and amplify inflammation with subsequent disease severity. Given the finding that the final levels of antibodies were similar in the mild and moderate/severe groups, we believe that the increased morbidity of the moderate/severe patient cohort is not due to differences in antibody function. In support of this notion, we further demonstrated the presence of potentially neutralizing antibodies in the serum of our moderate/severe patient cohort, which correlated with the levels of IgA and IgG. However, our assay can only detect the presence of antibodies that display neutralization potential towards RBD-ACE2 interaction since their actual neutralizing activity requires functional testing by blocking SARS-CoV-2 infection of epithelial cells. Nevertheless, it is important to note, that using the commercial anti-SARS-CoV-2 RBD neutralizing antibody, which was shown to block SARS-CoV-2 infection of epithelial cells at a 500 pg/ml, reach ~ 30% inhibition in our ACE2:RBD binding in our assay (Supplementary Fig. 5h). Furthermore, it should be noted that another possibility that out assay does not test is neutralization antibodies that are not directed against RBD. Thus, our neutralization method similar to other approaches^28,29^ add a great diagnostic value. Finally, our cytokine profiling analysis could not define a clear correlation between antibodies response in severe patients and proinflammatory cytokines as IL-1β, IL-6, IL-8 and TNF-α. However, it is possible that such a correlation will be identified in critically ill patients especially if cytokine expression will be monitored at the site of infection (i.e., lungs)^19^. Another explanation for the correlation that we observed between cytokines and antibody responses in moderate/severe patients in comparison to mild ones is supported by the recent publication that patients receiving cytokine inhibitors had lower prevalence of SARS-CoV-2 seroconversion^30^. Using hierarchical clustering analysis, we identified that IgG in the moderate/severe patient population clustered with IL-33. This is of specific interest since IL-33 is a cytokine that is released upon lung epithelial cell damage^31^, which is a marker of lung pathology, and suggested to play a role in IgG production during HIV-1 infection^32^.

Serological testing will serve in the near future as a powerful tool to conduct epidemiological studies in distinct populations and continents^8^. In addition to such studies, better understating the kinetics of anti-SARS-CoV-2 antibody responses will be critical in various therapeutic settings including utilizing antibodies as part of plasma/antibody therapy or alternatively monitoring the immune response in developing future vaccines. Although we did not monitor the biological function of the antibodies or the presence of long-lasting memory cells, our study demonstrates a clear reduction in IgA and IgM antibody levels starting at 57 days post symptoms. A trend toward reduction was also observed in IgG. In agreement, a slight reduction in neutralizing antibodies was observed. This is important since vaccines are largely based on generating long-lasting immunity and neutralizing IgG antibodies. Indeed, a recent publication revealed reduced levels of total and neutralizing antibodies between the acute phase and the convalescence phase^33^. It should be noted that the half-life of IgG (and not IgA nor IgM) is heavily dependent on the FcRn recycling system. Thus, when high levels of antibodies are reached, the recycling system is saturated, leading to fast reduction when pathogen is cleared and antigens are not available. Taken together with our results that IgG levels are still high for at least 120 days post symptoms and at least 90 days since negative PCR was obtained, suggest that vaccination should be achievable. If, following vaccination, a similar and slow reduction in IgG antibodies will be observed (similar to our findings), the exact vaccination regimen including secondary boosts for the generation of long-lasting memory should be considered.

In summary, our study provides a comprehensive analysis of the cytokine and antibody response, with specific emphasis on the kinetics of all three major antibody classes toward SARS-CoV-2 RBD and NP antigens in mild and moderate/severe patients and their neutralization potential. By establishing a rapid, quantitative, accurate, and robust method as well as analysis, our data have direct methodological implications for future clinical diagnostic, basic research and epidemiological surveys. In addition, our kinetic analysis provides important insights and considerations of future vaccination strategies.

## Materials and methods

### Reagents

Unless stated otherwise, all reagents were purchased from Biological Industries, Beit-Haemek, Israel. The SARS-CoV-2 receptor binding domain (RBD) antigen was homemade or purchased from Sigma-Aldrich (Cat. # SAE1000). SARS-CoV-2 nucleocapsid protein (NP) antigen was purchased from Aalto Bio Reagents (code CK 6404-b). ACE2-biotin (Cat. # SAE0171) was purchased from Sigma-Aldrich. HRP-conjugated secondary antibodies were purchased from Jackson ImmunoResearch Labs (West Grove, PA, USA). Regular ELISA plates were purchased from Greiner Bio-One. All reagents for the electrochemiluminescence test were purchased from MesoScale Diagnostic LLC: (MSD) MULTI-ARRAY 96 Plate Pack (Cat #: L15XA); Human/NHP IgG Detection Antibody Product (100 ug) (Cat #: D20JL); Human/NHP IgM Detection Antibody Product (100 μg) (Cat # D20JP); Human/NHP IgA Detection Antibody Product (100 μg) (Cat # D20JJ); MSD GOLD Read Buffer A (Cat #: R92TG); MSD Blocker A Kit (Cat #: R93AA). Sulfo-Tag streptavidin (Cat # R32AD-5). Anti-SARS-CoV-2 RBD Neutralizing Antibody, Human IgG1 was purchased from Acro biosystems (Cat # SAD-S35-100ug).

### Expression of recombinant SARS-CoV-2 RBD

The codon optimized sequence of SARS-CoV-2 RBD protein was synthesized by Syntezza-Israel and cloned into the pcDNA 3.1 mammalian expression vector. A hexa-histidine tag (his-tag) was added at the C-terminal for downstream protein purification. The construct was used to transiently transfect Expi293F cells (Thermo Fisher Scientific, Inc.) using the ExpiFectamine 293 Transfection Kit (Thermo Fisher Scientific, Inc.). Seven days post-transfection, the cell supernatant was collected, filtered (0.22 µm), and the protein was purified using Ni–NTA (GE Healthcare Life Sciences) affinity chromatography, washed and eluted using 250 mM imidazole. The RBD protein was buffer exchanged to PBS, aliquoted, and stored at − 80 °C.

### Patients and their sample collection

Patients’ samples were obtained from symptomatic individuals testing positive for SARS-Cov-2 by quantitative PCR. Samples were obtained from patients hospitalized at Hasharon Hospital, which is a designated Corona Hospital in Israel. In addition, samples were also obtained from anonymous patients who were tested positive for COVID-19 by RT-PCR. Peripheral blood was obtained (~ 5 ml) from each patient at different time points including during admission, hospitalization, dismissal, and/or during a routine check-up in the clinic for COVID-19 recovered patients. Samples were also obtained from blood bank donors; they were collected before November 2019. All experiments were reviewed and approved by the Ethics committee of the Rabin Medical Center, Beilinson Hospital (IRB#RMC-0265-20) and were performed according to their regulations and guidelines. Informed consent was obtained from all subjects.

### Disease severity definition

COVID-19 patients’ disease severity was defined for confirmed COVID-19 patients according to the Israel Ministry of Health as follows: (1) Mild disease—Respiratory disease in the upper airways or pneumonia that does not follow the stated definitions for moderate/severe disease; (2) Moderate disease—Pneumonia with one of the following characterizations (that does not follow the severe disease definitions): (a) More than 30 breaths per minute (RR > 30/min); (b) Respiratory distress; or (c) Less than 90% O_2_ saturation in room air; and (3) Severe disease—Pneumonia with a respiratory distress of RR > 30/min, blood oxygen saturation < 90%, respiratory failure [Acute respiratory distress syndrome (ARDS)], sepsis or shock.

### Serum preparation

Whole blood was centrifuged (500×*g*, 5 min) in secure buckets. Supernatant was transferred into a clean 1.7/2 ml Eppendorf tube. Thereafter, the serum was inactivated by heat (at 56 °C, for 30 min). The samples were apportioned into 50 μl aliquots and stored at − 20 °C or − 80 °C.

### TauMed ELISA protocol

Designated electrochemiluminescence plates were coated with 30 μl of purified antigen (RBD or NP at 2 μg/ml in PBS) and incubated overnight at 4 °C. Thereafter, the plates were washed three times (200 μl per well) with MSD washing buffer and blocked with 150 μl of MSD blocking buffer per well [1 h at Room Temp (RT)]. Blocking buffer was tapped out before adding 50 μl of the diluted sample to each well [3.6 μl of the sample was added to 176.4 μl of the sample diluent (PBST + 1% MSD blocker A)] and incubated for 30 min at RT. Subsequently, the plates were washed three times (200 μl per well) and 50 μl of detection antibodies were added and incubated for 20 min at RT. All of the detection antibodies were diluted in PBST + 1%MSD blocker A as follows: IgG detection antibodies to 0.25 μg/ml (1:2000); IgM detection antibodies to 0.25 μg/ml (1:2000); and IgA detection antibodies to 0.5 μg/ml (1:1000).

Finally, the plates were washed three times (200 μl per well) with MSD washing buffer, and 150 μl MSD Gold Read buffer was added to each well (avoiding air bubbles in each well). Plates were read within 20 min, using MESO QuickPlex SQ 120.

### Spotted TauMed ELISA protocol

RBD, at a concentration of 100 μg/ml (Cat. # SAE1000) was spotted by MSD on a MSD 10-spot 96-Well plate (Cat. # N05YA-1) according to their internal protocols and kept at 4 °C until use. Protocol for ELISA was done as above, starting at blocking stage. Detection antibodies were used as followed: IgG detection antibodies to 0.25 μg/ml (1:1000); IgM detection antibodies to 0.25 μg/ml (1:1000); IgA detection antibodies to 0.5ug/ml (1:1000). For neutralization assay, 50 μl of ACE2 biotin (0.2 pg/ml) and Sulfo-Tag streptavidin (0.1 ng/ml) were added to each well instead of the detection antibodies.

### HRP ELISA

HRP ELISA was performed similar to the above-mentioned protocol with the following changes: (1) blocking was conducted using 3% skim milk; (2) the sample incubation time was 2 h; (3) the detection antibody incubation time was 1 h; and (4) HRP substrate was added for 10 min and the reaction was stopped using 50 μl 1 N HCl. Readouts at 405 nm (using 595 nm background subtraction) were performed within 5 min using BioTek EPOCH2.

### Cytokine measurement

Cytokine were measure in the serum using the LEGENDplex Human Inflammation Panel 1 (13-plex) according to their protocol (10 μl of serum was diluted 1:2). At least 200 beads per cytokine were collected using ATTUNE NxT. Data was analyzed using LEGENDplex V. 8.0 software.

### Statistical analysis

All of the statistical analyses were performed using GraphPad Prism 8 software. Sensitivity and specificity were determined using ROC analysis (Wilson/Brown 95% CI). To compare ranks, a Nonparametric Mann–Whitney t-test was performed. In comparative assays, a one-way ANOVA nonparametric Kruskal–Wells test, followed by Dunn’s multiple comparisons test, was performed. In all experiments p values < 0.05 were considered significant. Hierarchical clustering of patients’ cytokine and seroconversion toward SARS-CoV2 RBD IgG/M/A antibodies was preformed using ClustVis^34^; Original values are ln(x + 1)-transformed. Rows are centered; unit variance scaling is applied to rows. Both rows and columns are clustered using correlation distance and complete linkage.

## Supplementary Information

Supplementary Information.

## References

[1] World Health Organization (WHO). *Novel Coronavirus (2019-nCoV) Situation Report—1 21 January 2020*. *WHO Bulletin* (2020).

[2] Li Q (2020). Early transmission dynamics in Wuhan, China, of novel coronavirus-infected pneumonia. N. Engl. J. Med..

[3] World Health Organization. Novel Coronavirus (COVID-19) Situation. *WHO (June 11)* (2020).

[4] Kontis V (2020). Magnitude, demographics and dynamics of the effect of the first wave of the COVID-19 pandemic on all-cause mortality in 21 industrialized countries. Nat. Med..

[5] Remuzzi A, Remuzzi G (2020). COVID-19 and Italy: What next?. Lancet (London, England).

[6] McKibbin WJ, Fernando R (2020). The global macroeconomic impacts of COVID-19: Seven scenarios. SSRN Electron. J..

[7] Li Y (2020). Stability issues of RT-PCR testing of SARS-CoV-2 for hospitalized patients clinically diagnosed with COVID-19. J. Med. Virol..

[8] Bryant JE (2020). Serology for SARS-CoV-2: Apprehensions, opportunities, and the path forward. Sci. Immunol..

[9] Long Q-X (2020). Antibody responses to SARS-CoV-2 in patients with COVID-19. Nat. Med..

[10] Chen Y (2020). A comprehensive, longitudinal analysis of humoral responses specific to four recombinant antigens of SARS-CoV-2 in severe and non-severe COVID-19 patients. PLoS Pathog..

[11] Krammer F, Simon V (2020). Serology assays to manage COVID-19. Science (80-)..

[12] Stowell SR, Guarner J (2020). Role of serology in the coronavirus disease 2019 pandemic. Clin. Infect. Dis..

[13] Tay MZ, Poh CM, Rénia L, MacAry PA, Ng LFP (2020). The trinity of COVID-19: Immunity, inflammation and intervention. Nat. Rev. Immunol..

[14] Zhao Z (2016). A multiplex assay combining insulin, GAD, IA-2 and transglutaminase autoantibodies to facilitate screening for pre-type 1 diabetes and celiac disease. J. Immunol. Methods.

[15] Dabitao D, Margolick JB, Lopez J, Bream JH (2011). Multiplex measurement of proinflammatory cytokines in human serum: Comparison of the Meso Scale Discovery electrochemiluminescence assay and the Cytometric Bead Array. J. Immunol. Methods.

[16] Amanna IJ, Carlson NE, Slifka MK (2007). Duration of humoral immunity to common viral and vaccine antigens. N. Engl. J. Med..

[17] Woo PCY (2005). Differential sensitivities of severe acute respiratory syndrome (SARS) Coronavirus spike polypeptide enzyme-linked immunosorbent assay (ELISA) and SARS coronavirus nucleocapsid protein ELISA for serodiagnosis of SARS coronavirus pneumonia. J. Clin. Microbiol..

[18] Jin, J.-M. *et al.* Gender differences in patients with COVID-19: Focus on severity and mortality. *Front. Public Health.* **8**, 152. 10.3389/fpubh.2020.00152 (2020). 10.3389/fpubh.2020.00152 PMC7201103 32411652

[19] Liao M (2020). Single-cell landscape of bronchoalveolar immune cells in patients with COVID-19. Nat. Med..

[20] Lucas C (2020). Longitudinal analyses reveal immunological misfiring in severe COVID-19. Nature.

[21] Grifoni A (2020). Targets of T cell responses to SARS-CoV-2 coronavirus in humans with COVID-19 disease and unexposed individuals. Cell.

[22] Perkmann, T. *et al.* Side by side comparison of three fully automated SARS-CoV-2 antibody assays with a focus on specificity. *medRxiv.* (2020). 10.1101/2020.06.04.20117911. 10.1093/clinchem/hvaa198 PMC7454460 32777031

[23] Yong SEF (2020). Connecting clusters of COVID-19: An epidemiological and serological investigation. Lancet Infect. Dis..

[24] Norman M (2020). Ultrasensitive high-resolution profiling of early seroconversion in patients with COVID-19. Nat. Biomed. Eng..

[25] Yongchen Z (2020). Different longitudinal patterns of nucleic acid and serology testing results based on disease severity of COVID-19 patients. Emerg. Microbes Infect..

[26] Pedersen SF, Ho Y-C (2020). SARS-CoV-2: A storm is raging. J. Clin. Investig..

[27] Tetro JA (2020). Is COVID-19 receiving ADE from other coronaviruses?. Microbes Infect..

[28] Tan CW (2020). A SARS-CoV-2 surrogate virus neutralization test based on antibody-mediated blockage of ACE2-spike protein–protein interaction. Nat. Biotechnol..

[29] Gattinger P (2020). Antibodies in serum of convalescent patients following mild COVID-19 do not always prevent virus-receptor binding. Allergy Eur. J. Allergy Clin. Immunol..

[30] Simon, D. *et al.* Patients with immune-mediated inflammatory diseases receiving cytokine inhibitors have low prevalence of SARS-CoV-2 seroconversion. *Nat. Commun.* **11**, (2020). 10.1038/s41467-020-17703-6 PMC7382482 32709909

[31] Shlomovitz I (2019). Necroptosis directly induces the release of full-length biologically active IL-33 in vitro and in an inflammatory disease model. FEBS J..

[32] Sarkar S (2019). IL-33 enhances the kinetics and quality of the antibody response to a DNA and protein-based HIV-1 Env vaccine. Vaccine.

[33] Long Q-X (2020). Clinical and immunological assessment of asymptomatic SARS-CoV-2 infections. Nat. Med..

[34] Metsalu T, Vilo J (2015). ClustVis: A web tool for visualizing clustering of multivariate data using Principal Component Analysis and heatmap. Nucleic Acids Res..

