# Supplementary material for: Rapid seroconversion and persistent functional IgG antibodies in severe COVID-19 patients correlates with an IL-12p70 and IL-33 signature
Source: Sci Rep. 2021 Feb 10;11:3461. doi: 10.1038/s41598-021-83019-0 (PMC7876035; doi:10.1038/s41598-021-83019-0)
Supplement: Supplementary file 1 — Supplementary Information. [file 41598_2021_83019_MOESM1_ESM.pdf]

Supplementary information

Rapid seroconversion and persistent functional IgG antibodies in severe COVID-19 patients correlates with an IL-12p70 and IL-33 signature

Munitz A[1,*], Edry-Botzer L[1], Itan M[1], Tur-Kaspa R[2], Dicker D[3], Marcoviciu D[3], Goren GM[1], Mor M[1], Lev S[4], Gottesman T[5], Muhsen K[6], Cohen D[6], Stein M[7], Qimron U[1], Freund NT[1], Wine Y[8], and Gerlic M[1, *].

[1] Department of Clinical Microbiology and Immunology, Sackler School of Medicine, Tel Aviv University, 6997801 Israel

[2] Department of Medicine D and the Liver Institute, Rabin Medical Center, Beilinson Hospital, Molecular Hepatology Research Laboratory, Felsenstein Medical Research Center Sackler School of Medicine Tel-Aviv University, Faculty of Medicine Bar-Ilan University, Liver Institute Rabin Medical Center Beilinson Hospital Petach-Tikva Israel 39100

[3] Internal Medicine D, Hasharon Hospital-Rabin Medical Center, Petach Tikva, Israel. Sackler School of Medicine, Tel Aviv University, 6997801 Israel

[4] Intensive care unit, Hasharon Hospital-Rabin Medical Center, Petach Tikva, Israel. Sackler School of Medicine, Tel Aviv University, 6997801 Israel

[5] Infectious diseases and infection control, Hasharon Hospital-Rabin Medical Center, Petach Tikva, Israel. Sackler School of Medicine, Tel Aviv University, 6997801 Israel

[6] Department of Epidemiology and Preventive Medicine, School of Public Health, Tel Aviv University, 6997801 Israel

[7] Allergy and Clinical Immunology Unit, Wolfson Medical Center, 6997801 and Sackler School of Medicine, Tel Aviv University, Israel

[8] The Shmunis School of Biomedicine and Cancer Research, The George S. Wise Faculty of Life Sciences, Tel Aviv University, 6997801 Israel

Supplemental Tables

Supplementary Table 1 – Hospitalized patient characteristics

Disease severity	Gender	N=	Age (years)	DPS (Days)	Lymphocyte count (10^6/ml)	CRP (mg/L)
Mild (N=29)	Female	13	66.58 ± 19.07	18.08 ± 18.64	1.75 ± 0.63	0.74 ± 0.66
	Male	16	65.41 ± 14.68	29.75 ± 23.73	1.4 ± 0.63	5.66 ± 4.78
Moderate /Severe (N=28)	Female	5	76.20 ± 4.71	16.00 ± 14.68	2.70 ± 4.36	6.96 ± 5.37
	Male	23	67.16 ± 15.36	21.22 ± 16.14	1.64 ± 2.10	5.10 ± 7.76

Supplementary Table 2- anti RBD antigen

Anti RBD IgG		
Days post symptoms onset (n)	>2671 RLU (Specificity 94.87%, 95% CI, n=195)	>3830 RLU (Specificity 97.95%, 95% CI, n=195)
	Sensitivity	Sensitivity
1-7 (n=13)	46.15%	46.15%
8-14 (n=15)	66.67%	66.67%
>14 days (n=68)	92.65%	88.24%
Total (n=96)	82.29%	79.17%
Anti-RBD IgM		
Days post symptoms onset (n)	>2707 RLU (Specificity 94.87%, 95% CI, n=195)	>3878 RLU (Specificity 97.95%, 95% CI, n=195)
	Sensitivity	Sensitivity
1-7 (n=13)	53.85%	46.15%
8-14 (n=15)	53.33%	40.00%
>14 days (n=68)	95.59%	91.18%
Total (n=96)	83.33%	77.08%
Anti-RBD IgA		
Days post symptoms onset (n)	>709.3 RLU (Specificity 94.85%, 95% CI, n=97)	>906.3 RLU (Specificity 97.94%, 95% CI, n=97)
	Sensitivity	Sensitivity
1-7 (n=13)	61.54%	61.54%
8-14 (n=15)	86.67%	73.33%
>14 days (n=68)	91.18%	77.94%
Total (n=96)	86.46%	75.00%

In some patients more than one sample (different time points) was collected. This cohort also includes anonymous recovered patients which are not in Supplementary Table 1.

Supplementary Table 3 – Anti-RBD combined analysis using 98% specificity for each individual antibody

Days post symptoms onset (n)	Negative	IgG	IgM	IgA	IgA & IgG	IgM & IgA	IgM & IgG	IgM & IgG & IgA	Sensitivity
1-7 (n=13)	2	1	0	4	0	1	2	3	84.6%
8-14 (n=15)	3	0	0	2	4	0	1	5	80.0%
>14 days (n=68)	0	2	4	0	4	4	9	45	100.0%
Total (n=96)	5	3	4	6	8	5	12	53	94.8%

- In some patients more than one sample (different time points) was collected. This cohort also includes anonymous recovered patients which are not in Supplementary Table 1.

Supplementary Table 4 – Anti-NP antigen

Anti NP IgG		
Days post symptoms onset (n)	>1245 RLU (Specificity 95.56%, 95% CI, n=90)	>1995 RLU (Specificity 97.78%, 95% CI, n=90)
	Sensitivity	Sensitivity
1-7 (n=13)	53.85%	53.85%
8-14 (n=14)	64.29%	57.14%
>14 days (n=31)	96.77%	96.77%
Total (n=58)	79.31%	77.59%
Anti-NP IgM		
Days post symptoms onset (n)	>5982 RLU (Specificity 95.56%, 95% CI, n=90)	>7466 RLU (Specificity 97.78%, 95% CI, n=90)
	Sensitivity	Sensitivity
1-7 (n=13)	0%	0%
8-14 (n=14)	28.57%	28.57%
>14 days (n=31)	32.26%	32.26%
Total (n=58)	24.14%	24.14%
Anti-NP IgA		
Days post symptoms (n)	>1891 RLU (Specificity 95.56%, 95% CI, n=90)	>4500 RLU (Specificity 97.78%, 95% CI, n=90)
	Sensitivity	Sensitivity
1-7 (n=13)	15.38%	0%
8-14 (n=14)	57.14%	57.14%
>14 days (n=31)	87.10%	54.84%
Total (n=58)	63.79%	43.10%

- In some patients more than one sample (different time points) was collected. This cohort also includes anonymous recovered patients which are not in Supplementary Table 1.

Supplementary Table 5 – Anti-NP combined analysis using 98% specificity for each individual antibody

Days post symptoms (n)	Negative	IgG	IgM	IgA	IgA & IgG	IgM & IgA	IgM & IgG	IgM & IgG & IgA	Sensitivity
1-7 (n=13)	6	7	0	0	0	0	0	0	53.8%
8-14 (n=14)	5	1	0	1	3	0	0	4	64.3%
>14 days (n=31)	1	11	0	0	9	0	2	8	96.8%
Total (n=58)	12	19	0	1	12	0	2	12	79.3%

- In some patients more than one sample (different time points) was collected. This cohort also includes anonymous recovered patients which are not in Supplementary Table 1.

Supplementary Table 6 – Anti-RBD and anti-NP IgGs combined analysis using 100% specificity for each individual antibody

Days post symptoms onset (n)	Negative	Anti-RBD	Anti-NP	Anti-RBD & anti-NP	Sensitivity
1-7 (n=13)	11	0	0	2	15.4%
8-14 (n=14)	7	0	0	7	50.0%
>14 days (n=31)	1	0	6	24	96.8%
Total (n=58)	19	0	6	33	67.2%

- In some patients more than one sample (different time points) was collected. This cohort also includes anonymous recovered patients which are not in Supplementary Table 1.

Supplemental Figures

Supplemental Figure 1

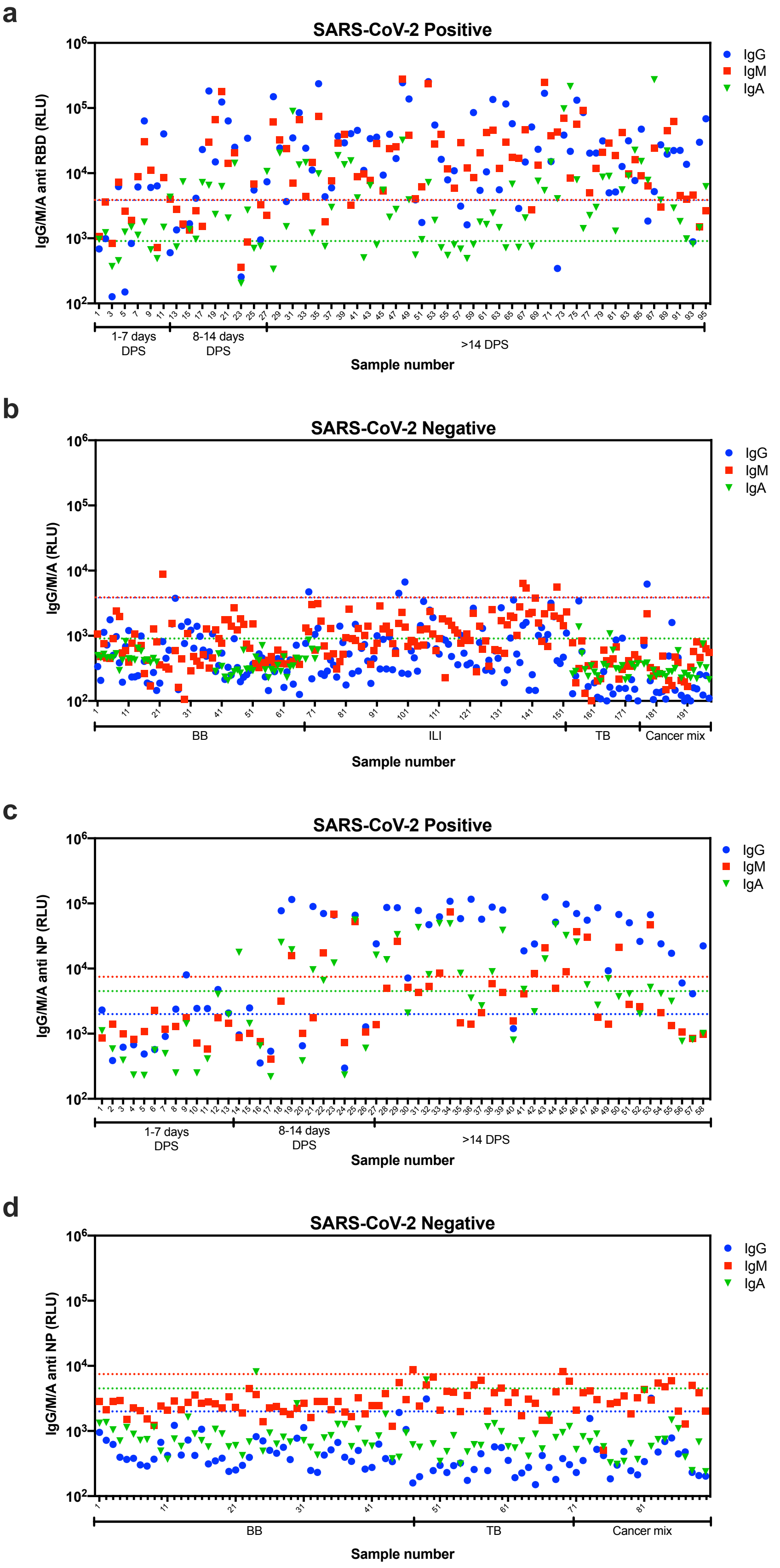

Supplementary Figure 1. Individual anti-SARS-CoV-2-RBD and -NP antibodies using electrochemiluminescence ELISA. Peripheral blood was collected from the peripheral blood of hospitalized COVID-19 patients and anonymous recovered patients. Negative samples were obtained from true SARS-CoV-2 negative patients (i.e., prior to the SARS-CoV-2 pandemic) (n=197). Plasma was obtained, diluted 1:50, and added to a 96-well plate precoated with SARS-CoV-2 RBD antigen. Individual IgG (blue), IgM (red), and IgA (green) levels of each SARS-CoV-2 positive (n=96 and n=58 for RBD and NP respectively) (a, c) and a negative sample (n=197 and n=90 for RBD and NP respectively) (b, d) are shown. Data were calculated using GraphPad Prism 8; the dotted line represents the calculated cutoff value discriminating between positive and negative samples.

Supplemental Figure 2

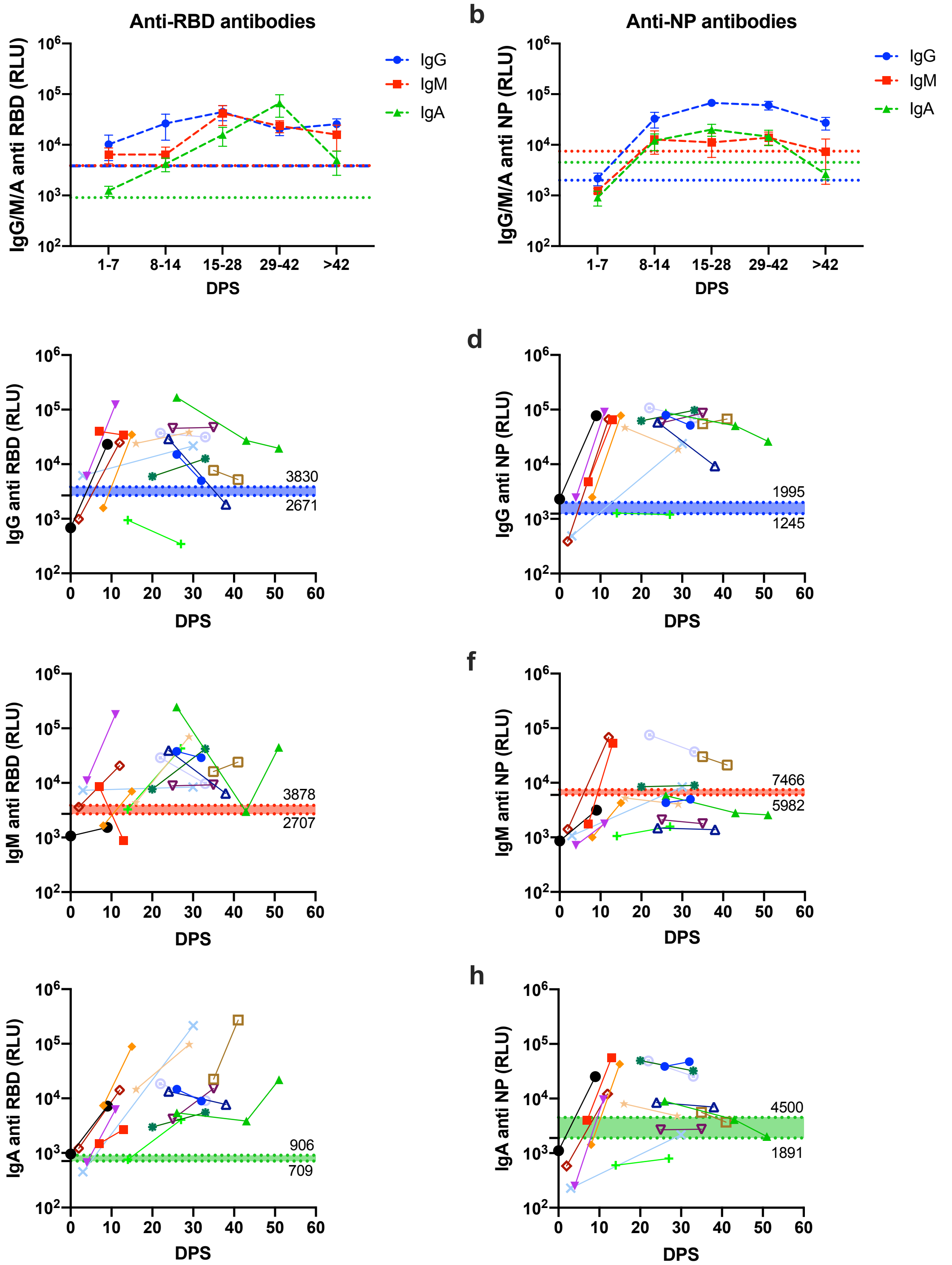

Supplementary Figure 2. SARS-CoV-2 antibodies' response kinetics. Peripheral blood was collected from hospitalized COVID-19 patients. Plasma was obtained, diluted 1:50, and added to a 96-well plate precoated with SARS-CoV-2 RBD (a, c, e, g) or NP (b, d, f, h) antigens. a, b Kinetics of all samples; average±SEM; n=57. c-h Kinetics of individual patient's antibody response. Data were calculated using GraphPad Prism 8; the dotted line represents the calculated cutoff values (98% sensitivity for a, b, and 95% and 98% sensitivity for c-h) discriminating between positive and negative samples.

Supplemental Figure 3

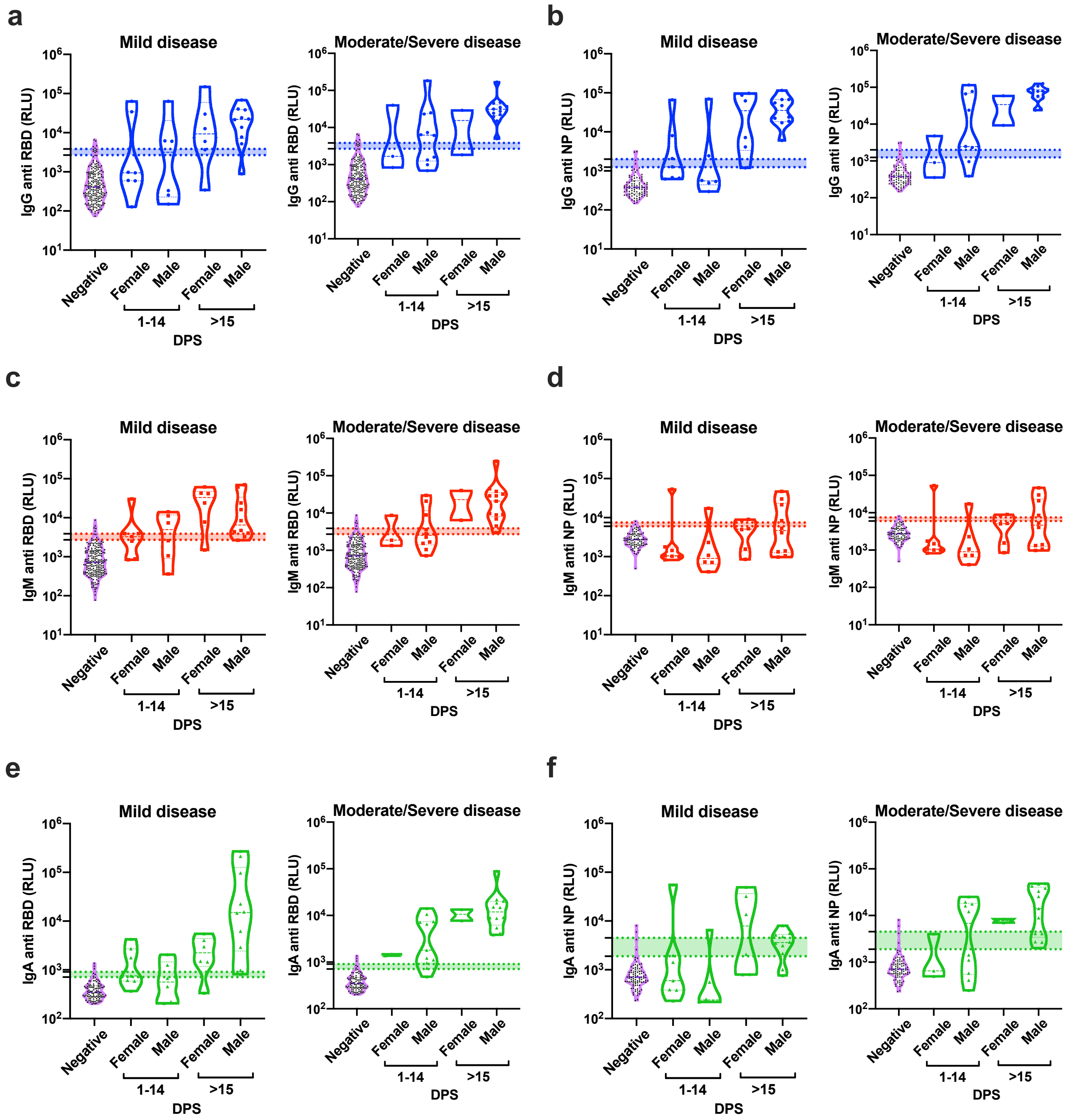

Supplemental Figure 3. No correlation of antibodies' response to patient gender. Peripheral blood was collected from hospitalized COVID-19 patients. Negative samples were obtained from true SARS-CoV-2 negative patients (i.e., prior to the SARS-CoV-2 pandemic). Plasma was obtained, diluted 1:50, and added to a 96-well plate precoated with SARS-CoV-2 RBD (a, c, e) or NP (b, d, f) antigens. Patients'

antibody results were grouped according to their gender and the disease severity and graphed against DPS (1-14 and >15). Data were calculated using GraphPad Prism 8; the dotted line represents the calculated cutoff values (95% and 98% sensitivity) discriminating between positive and negative samples. Statistical analysis was performed using a Nonparametric Kruskal-Wells test for multiple comparisons against between female and male groups. No significant difference was found between genders.

Supplemental Figure 4

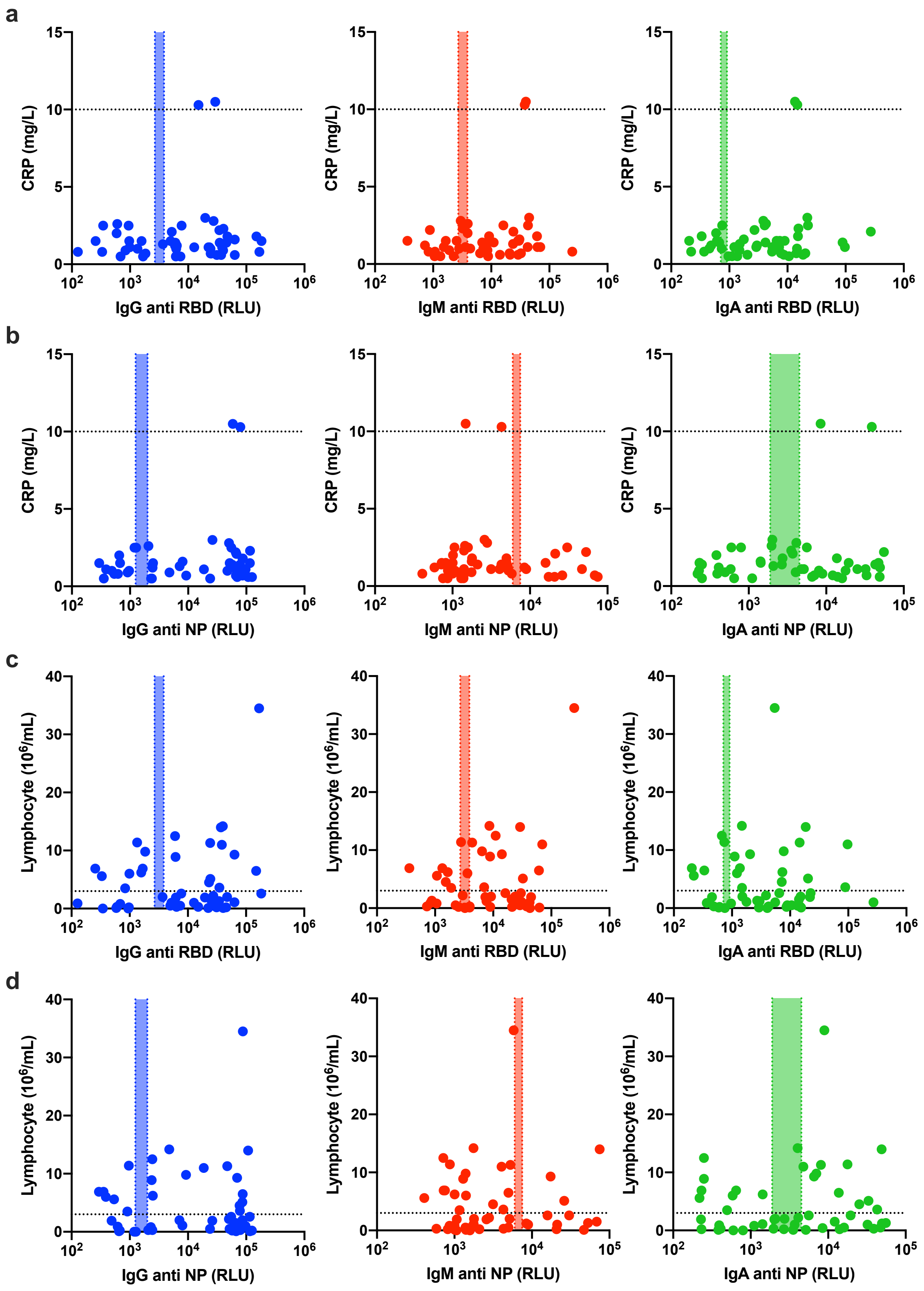

Supplemental Figure 4. No correlation of antibodies' response to Lymphocyte count nor to the CRP levels. Peripheral blood was collected from hospitalized COVID-19 patients. Lymphocyte count and CRP levels were determined immediately. Plasma was obtained, diluted 1:50, and added to a 96-well plate precoated with SARS-CoV-2 RBD (a, c) or NP (b, d) antigens. Patients' antibody results were graphed against the CRP levels (a-b) and the lymphocyte count (c-d). Data were calculated using GraphPad Prism 8; the dotted X-line represents the calculated cutoff values (95% and 98% sensitivity) discriminating between positive and negative samples, whereas the dotted Y-line represents the cutoff of high levels of either CRP (>10mg/L) or the lymphocyte count (>$3x10^6$/mL). Correlation analysis was performed using a nonparametric Spearman's correlation test (two-tailed, 95% confidence). No correlation was found.

Supplemental Figure 5

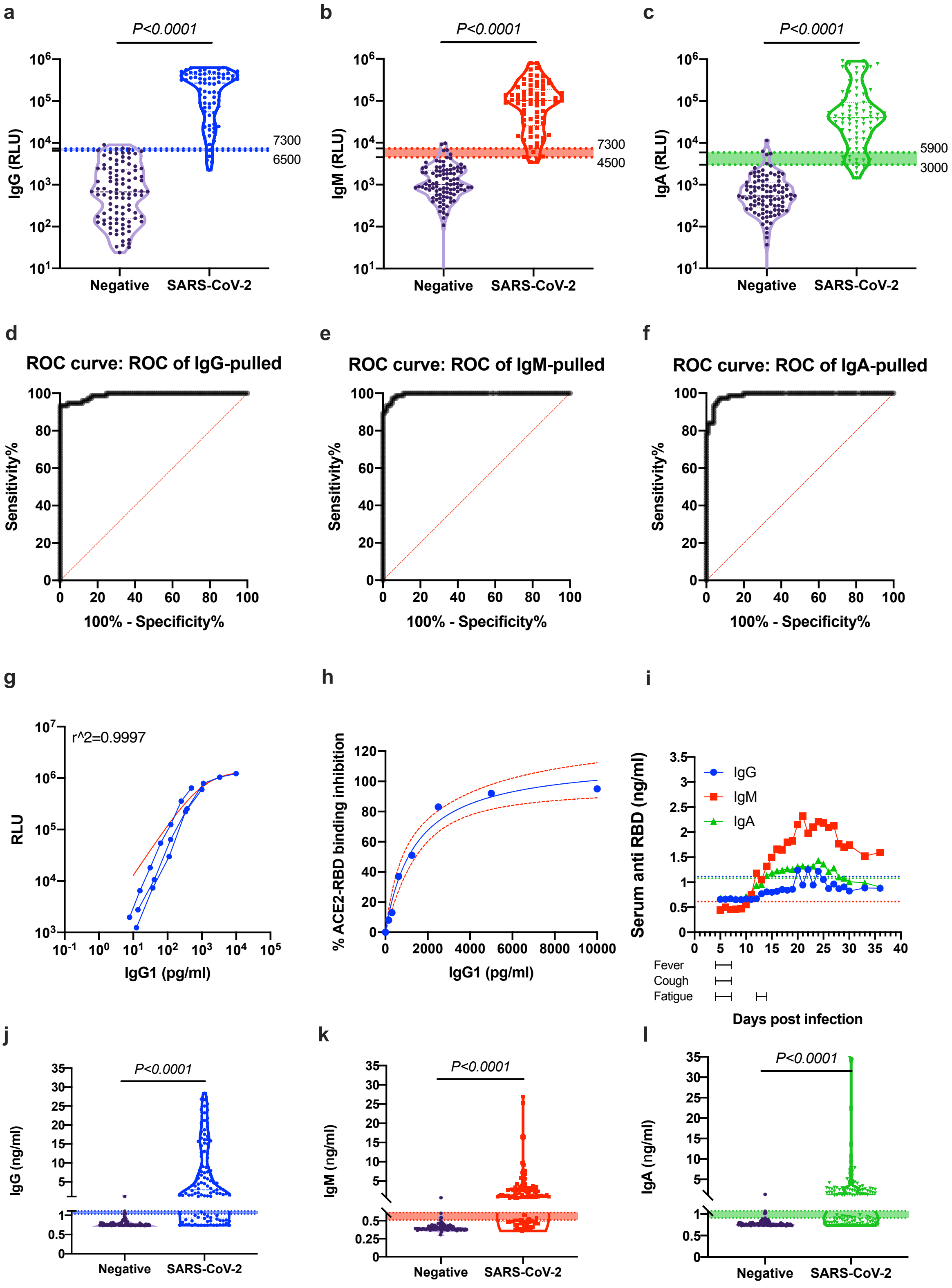

Supplemental Figure 5. Validation of anti-SARS-CoV-2-RBD antibodies using spotted electrochemiluminescence ELISA. Peripheral blood was collected from the hospitalized peripheral blood of COVID-19 and anonymous recovered patients (n=75). Negative samples were obtained from true SARS-CoV-2 negative patients (i.e., prior to the SARS-CoV-2 pandemic) (n=101). Plasma was obtained, diluted 1:50, and added to a 10-spot 96-well plate spotted with SARS-CoV-2 RBD antigen on spot number 1, and BSA on spots number 2-10. IgG (a), IgM (b), and IgA (c) levels as well as ROC analysis (d-f) are shown. g, Interpolation of IgG1 standard using spotted plates is shown. h, %ACE2-RBD inhibition was graphed against known amount of neutralizing IgG1 antibody. i, Kinetic of quantitative antibody response for individual patient. j-l, Quantitative antibody response for all patients (0-122 DPS) using RBD spotted plate is shown. Data were calculated using GraphPad Prism 8; the dotted line represents the calculated cutoff value discriminating between positive and negative samples. (a-c, j-l) A nonparametric Mann-Whitney t-test was performed. P values are shown.
